# Primary pulmonary synovial sarcoma requiring differentiation from pulmonary metastasis of tibial adamantinoma: a case report

**DOI:** 10.1186/1756-0500-7-736

**Published:** 2014-10-18

**Authors:** Toshiharu Shirai, Shinji Tsuchida, Ryu Terauchi, Naoki Mizoshiri, Eiichi Konishi, Yasuhiko Tomita, Junichi Shimada, Hiroyoshi Fujiwara, Toshikazu Kubo

**Affiliations:** Department of Orthopaedics, Graduate School of Medical Science, Kyoto Prefectural University of Medicine, Kawaramachi-Hirokoji, Kamigyo-ku, Kyoto, 602-8566 Japan; Department of Orthopaedic Surgery, Graduate School of Medical Science, Kanazawa University, 13-1 Takara-machi, Kanazawa, 920–8641 Japan; Department of Surgical Pathology, Graduate School of Medical Science, Kyoto Prefectural University of Medicine, Kawaramachi-Hirokoji, Kamigyo-ku, Kyoto, 602-8566 Japan; Department of Pathology, Osaka Medical Center for Cancer and Cardiovascular Diseases, 1-3-3 Nakamichi, Higashinari-ku, Osaka, 537-8511 Japan; Department of General Thoracic Surgery, Graduate School of Medical Science, Kyoto Prefectural University of Medicine, Kawaramachi-Hirokoji, Kamigyo-ku, Kyoto, 602-8566 Japan

**Keywords:** Primary pulmonary synovial sarcoma, Adamantinoma, Pulmonary bone tumor metastasis

## Abstract

**Background:**

Primary pulmonary synovial sarcoma (PPSS) is rare. We describe a case of PPSS complicated by tibial adamantinoma that required differentiation from lung metastasis.

**Case presentation:**

A 39-year-old Japanese woman presented with hemoptysis, dyspnea, and a well-defined tumor measuring 3.0 cm in greatest diameter in the right lower lobe on chest computed tomography (CT). Positron emission tomography/CT with fluorodeoxyglucose (FDG-PET/CT) showed mild uptake of FDG (maximum standardized uptake value of 2.0). Her past history included surgery for adamantinoma of the right tibia at age 25 years. We considered the possibility of pulmonary metastasis from the adamantinoma and performed fluoroscopy-assisted thoracoscopic resection of the tumor after CT-guided Lipiodol marking. Histologically, the tumor was composed mainly of a dense proliferation of spindle cells. Immunohistochemical studies were positive for epithelial membrane antigen, B cell lymphoma 2, and transducing-like enhancer of split 1. They were negative for CD34. The *synovial sarcoma, X breakpoint 1* gene-fusion transcript was detected by reverse transcription-polymerase chain reaction. It is diagnostic of PPSS. Resection margins were negative. The patient was well without evidence of recurrence or metastasis of the PPSS or adamantinoma at the 30-month and 15-year follow-ups.

**Conclusion:**

Clinical and radiological manifestations of PPSS overlap with those of other lung tumors. The solitary pulmonary nodule in this case was indistinguishable from pulmonary metastases of the adamantinoma based on clinical symptoms, epidemiology, chest radiography, CT, and FDG-PET/CT. PPSS was diagnosed only after evaluating gross pathology, histology, immunohistochemistry, and cytogenetics. PPSS should be included in the differential diagnosis of a well-defined homogeneous round or oval lung mass. To our knowledge, this is the first report of PPSS complicated by adamantinoma.

## Background

Most lung tumors are malignant, carcinomatous metastases from other tissue cancers, such as bone tumors. Primary lung sarcoma is rare, accounting for less than 0.5% of all lung tumors [1, 2]. The variety of soft tissue sarcomas reflects the range of mesenchymal tissues present in the lung. These sarcomas include extraskeletal Ewing’s sarcoma, malignant fibrous histiocytoma, and synovial sarcoma. The latter is a rare sarcoma accounting for approximately 8% of soft tissue sarcomas. Although synovial sarcoma predominantly affects the extremities of young adults, primary lesions have also been identified in the head and neck, abdominal wall, and thorax (including the mediastinum, pericardium, pleura, and lung) [3–5]. A recent MEDLINE search revealed approximately 92 cases of primary pulmonary synovial sarcoma (PPSS) reported worldwide during the last three and a half decades. There were no reports, however, of PPSS complicated by a primary bone tumor. Here we report a woman with PPSS that required differentiation from tibial adamantinoma metastasis.

## Case presentation

### Initial presentation and diagnostics

**Figure 1:**
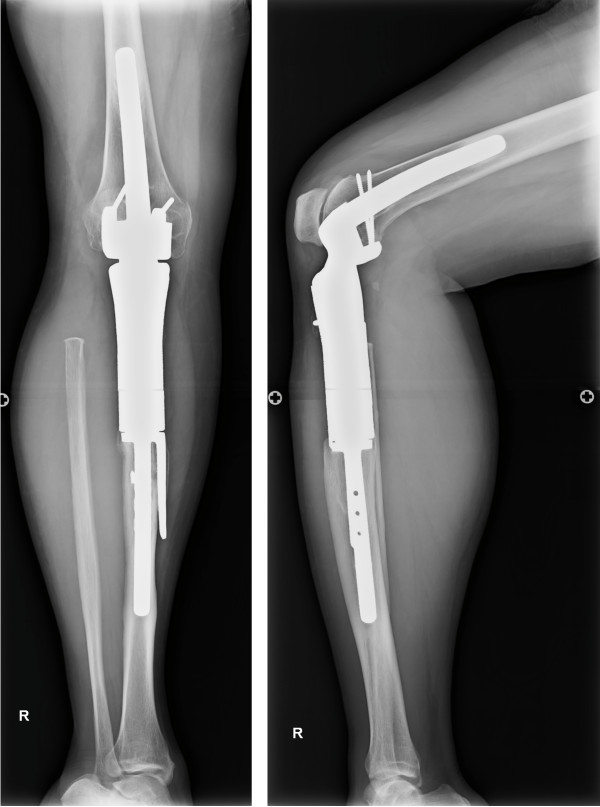
**Postoperative radiographs of tibial adamantinoma.** Anteroposterior and lateral radiographs show the uncemented megaprothesis (Howmedica Modular Replacement System) used to treat the patient after prior resection of an adamantinoma of the right tibia. No evidence of recurrence is present.

**Figure 2:**
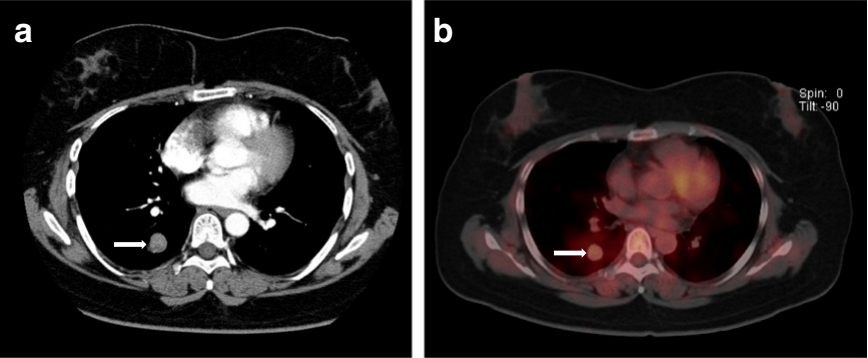
**Preoperative computed tomography and positron emission tomography with fluorodeoxyglucose.** Axial section of computed tomography **(a)** and positron emission tomography/computed tomography with fluorodeoxyglucose **(b)** show slightly high uptake by the right lung mass (arrow).

A 39-year-old Japanese woman, who was a household helper, presented to a community hospital with hemoptysis and dyspnea. Other symptoms, such as cough and chest pain, were not presented. She reported a family history of stomach cancer (her father). She had smoked one or two cigarettes daily for the last 19 years. There was no history of asbestos exposure or tuberculosis. She had undergone wide resection of an adamantinoma of the right tibia and limb salvage with an uncemented megaprosthesis (Howmedica Modular Replacement System; Howmedica International, Limerick, Ireland) 15 years previously (Figure 1). Her vital signs and clinical examination results were unremarkable. A general survey revealed no clubbing and no palpable cervical or axillary lymph nodes. Sputum was negative for acid-fast bacilli. The serum tumor marker levels were within their respective normal ranges.Chest radiography demonstrated a mass in the right lower field without pleural effusion. Computed tomography (CT) of the lung revealed a mass measuring 2.5 × 3.0 cm that had no contact with the pleura, main bronchus, inferior pulmonary vein, or right atrium (Figure 2a). Bronchoscopy did not reveal any abnormalities. Positron emission tomography (PET)/CT with fluorodeoxyglucose (FDG) showed mild FDG uptake, with a maximum standardized uptake value (SUVmax) of 2.0 (Figure 2b).

### Treatment

Because the tumor was suspected to be a pulmonary metastasis from the earlier adamantinoma, we surgically resected it without prior biopsy. The procedure was as follows. The pulmonary nodule was marked with 0.4 mL of Lipiodol® (Laboratoire Guerbet, Roissy, France) under CT guidance. The visceral pleura near the nodule was marked with 1 mL of a mixture of atelocollagen and methylene blue. During the thoracoscopic surgery performed 1 day later, the marked nodule was grasped with ring-shaped forceps under C-arm fluoroscopic guidance and resected [6, 7].

### Histology

**Figure 3:**
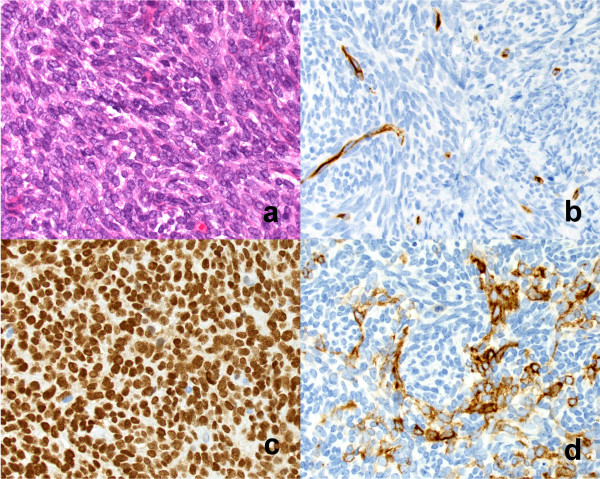
**Histology. (a)** Photomicrograph shows histopathological features of spindle cell sarcoma (H&E, ×20). **(b-d)** Photomicrograph shows spindle cells positive for the presence of CD34, transducin-like enhancer of split 1, and pancytokeratin.

Histopathologically, the tumor showed densely proliferated spindle cells with a high mitosis rate (Figure 3a). The immunohistochemical workup revealed that the spindle cell component was positive for B cell lymphoma 2 (BCL-2), pancytokeratin (MNF116) directed to cytokeratins 5, 6, 8, 17, and 19, transducin-like enhancer of split 1 (TLE-1), and epithelial membrane antigen (EMA). It was negative for CD34 (Figure 3b-d). The synovial *sarcoma, X breakpoint 1* (*SYT-SSX1*) fusion gene was detected by reverse transcription-polymerase chain reaction using ribonucleic acid samples from the tumor tissue. Hence, the final diagnosis was PPSS. Resection margins were negative.

### Follow-up

The patient’s hemoptysis and dyspnea resolved after the surgical procedure. Because the patient had factors indicating a poor PPSS prognosis, including her age (>20 years), sex, and the presence of the *SYT-SSX1* variant gene, we recommended chemotherapy with a combination of ifosfamide and doxorubicin. She refused further treatment, however. Follow-up visits were undertaken every 3 months after the surgery. She has remained well without evidence of recurrence or metastasis of the PPSS or the adamantinoma at the 30-month follow-up.

## Discussion

Soft tissue synovial sarcoma is a clinicopathologically and cytogenetically distinctive neoplasm that mainly affects deep soft tissues of the extremities in adolescents and young adults. Synovial sarcoma is also recognized as a primary pulmonary neoplasm [8]. PPSS is an aggressive tumor whose histological features are also seen in soft tissue synovial sarcoma [9]. PPSS occurs in older patients as well, however, in contrast to other soft tissue synovial sarcomas, with patients’ ages ranging from 9 to 84 years (mean 43 years) without sex bias [3–5]. Hartel *et al.* [10] reported that clinical symptoms are dyspnea (42%), chest pain (39%), cough (32%), and hemoptysis (22%). There are few asymptomatic cases. Most PPSSs are located in the lung parenchyma (58%), pleura (33%), and mediastinum (9%). Rarely, they extend into the bronchial tree or occur in the heart or pericardium [3, 4, 8, 11]. PPSS lesions range in size from 0.6 to 27.0 cm (mean 6.8 cm) [4, 10]. Radiologically, in contrast to soft tissue synovial sarcoma, PPSS is typically uniform with well-circumscribed rounded or lobulated borders [12, 13]. The contralateral lung usually appears normal, although mediastinal shift may occur in the presence of an extremely large mass [11]. CT demonstrates a well-defined homogeneous or heterogeneously enhancing mass containing necrotic areas and soft tissue components with common ipsilateral pleural effusion [11, 12]. Similar to all sarcomas, metastasis occurs via the blood, and adenopathy is uncommon. Endobronchial invasion is also not common, unlike lung carcinoma [11, 14]. Because of their high metabolic activity, these lesions show increased FDG uptake (SUVmax of >  2.5) on PET/CT [15].

Adamantinoma, in contrast, is a low-grade neoplasm that affects long bones and accounts for less than 1% of all primary bone tumors. These lesions occur most commonly in patients 10–50 years of age with slight male preponderance [16, 17]. They are locally aggressive. The incidence of recurrence is approximately 30%, and that of metastases ranges between 10% and 20% [16, 17]. They are capable of sending off distant metastases, especially to the lung [18]. It is not uncommon to develop pulmonary metastases even up to 10 years after detection of the primary lesion [19]. Radiologically, the tumor is typically uniform, well circumscribed, and similar to other pulmonary metastases of bone tumors. Pulmonary metastases of adamantinomas range in size from 0.4 to 10.0 cm (mean 4.7 cm) [16–19].

We considered pulmonary metastasis of adamantinoma in the differential diagnosis of our case because of the CT and FDG-PET/CT findings, the past history of adamantinoma, and the lack of reports of PPSS in conjunction with another tumor in the literature. Metastatectomy to remove pulmonary adamantinoma lesions with good results has been reported [19]. There appears to be no definitive role for radiotherapy or chemotherapy [20]. We therefore performed fluoroscopy-assisted thoracoscopic resection of the small pulmonary tumor in our patient. Unexpectedly, the final diagnosis based on histological findings and cytogenetic analysis was PPSS. Matsuo *et al.* [21] concluded that, although rare, PPSS should be included in the differential diagnosis of a well-defined homogeneous, round lung mass on CT images. However, the clinical and radiological manifestations of PPSS overlap with those of other lung lesions, including malignant melanoma, epithelioid schwannoma, malignant fibrous histiocytoma, malignant mesothelioma, adenocarcinoma, carcinosarcoma, lung hydatid disease, metastatic carcinoma, bronchogenic carcinoma, mesothelioma, lymphoma, Wegener’s granulomatosis, pyogenic abscess, intrapulmonary hematoma, rheumatoid nodules, histoplasmosis, coccidioidomycosis, and bone tumor metastasis [15, 22]. These primary lung lesions are typically indistinguishable from PPSS based on clinical symptoms, epidemiology, chest radiography, CT, and FDG-PET/CT [12, 23]. To reach a correct diagnosis, the t(X; 18)(p11.2;q11.2) chromosomal translocation should be demonstrated by cytogenetic testing. This analysis is the only primary test for diagnosing synovial sarcoma [9, 15]. According to Akerman *et al.* [24], the diagnosis of synovial sarcoma in large lesions (>3 cm) may be suggested in fine-needle aspiration biopsy specimens, but adjunctive methods are necessary for a definitive diagnosis. As a result, it may be that only after surgery is it possible to diagnose a small PPSS that has no contact with other internal organs, as in our case.

Because of its rarity and the paucity of data regarding its natural history, there are no guidelines regarding the optimal treatment for PPSS. Current treatment thus includes surgical resection (lobectomy or pneumonectomy) [25]. In our case, complete surgical excision of PPSS was performed by fluoroscopy-assisted thoracoscopic resection. Patient age, a positive surgical margin, and tumor size help guide the decision about whether additional adjuvant chemotherapy and/or radiation therapy is needed [25]. The overall 5-year survival rate for those with PPSS is 50%. Factors that indicate a poor prognosis include age >20 years, female sex, tumor size >5 cm, positive resection margin, extensive tumor necrosis, high number of mitoses (>10 per 10 high-power fields), neurovascular invasion, and the presence of the *SYT-SSX1* variant gene [25]. Although our patient had several factors that put her at risk of a poor prognosis, she experienced no recurrence during 30 months of postoperative follow-up.

We had recommended that our patient undergo chemotherapy with ifosfamide and doxorubicin, but she refused further treatment. We also suggested pazopanib, which interferes with vascular endothelial growth factor and platelet-derived growth factor pathways. Pazopanib is the first nonchemotherapeutic anticancer agent approved by regulatory authorities for soft tissue sarcoma and to which pulmonary adamantinoma metastasis responds [26, 27]. Long-term follow-up is necessary for patients with a soft tissue sarcoma.

## Conclusions

This is the first report of PPSS complicated by tibial adamantinoma, which is a low-grade malignant primary bone tumor. PPSS is a rare tumor without specific symptoms, signs, or radiological features. The role of functional imaging has not yet been evaluated, and the diagnosis is usually based on histopathological examination or cytogenetic testing of the resected tumor. The diagnosis of PPSS before surgery also may not be possible, as in our patient. Along with pulmonary metastasis from a bone tumor, PPSS should be included in the differential diagnosis of a well-defined homogeneous round or oval lung mass.

## Consent

Written informed consent was obtained from the patient for publication of this Case Report and any accompanying images. A copy of the written consent form from our university hospital is available for review by the Editor-in-Chief of this journal.

## Abbreviations

PPSS: Primary pulmonary synovial sarcoma
CT: Computed tomography
PET: Positron emission tomography
FDG: Fluorodeoxyglucose
SUVmax: Maximum standardized uptake value
BCL-2: B cell lymphoma 2
MNF116: Pancytokeratin
TLE-1: Transducin-like enhancer of split 1
EMA: Epithelial membrane antigen
*SYT-SSX1*: *Synovial sarcoma, X breakpoint 1*.
